# Employees and the National Insurance Act

**Published:** 1912-07-06

**Authors:** 


### NATIONAL INSURANCE ACT DEPARTMENT.

### EMPLOYEES AND THE NATIONAL INSURANCE ACT.

### XVIII.—Important Points for Immediate Consideration.

In little more than a week the National Insurance Act, 1911, will come into operation, and within a few days from July 15 a large proportion of the mighty host of insured persons will have experienced its first effect by the deduction from their wages of their contributions, while the employers will have had to purchase stamps and affix them to insurance cards.

It is, perhaps, as well that we should point out that a misconception has arisen in certain quarters through an incorrect reading of an official statement to the effect that it is not essential that the insured person should choose his approved society until October 13 next. We have found that this has been taken by some people to mean that they can, if they like, obtain a further three months' concession before they need enter into insurance, and that they will not be obliged to pay the contributions until that time. This is, of course, an inaccuracy. Every "employed person" who comes within the meaning of that term as defined in the Act must be insured as from July 15, but the insured person need not choose the particular approved society through which to receive the benefits until the later date. There are no benefits to receive for the first six months, with the single exception of sanatorium benefit, and consequently there is no immediate necessity so far as benefits are concerned in joining an approved society.

#### Reasons for Joining an Approved Society at Once.

On the other hand, there are very substantial reasons why the choice of a society should not be deferred. In the first place, every approved society has the power to refuse admittance to any applicant on any ground, except that of age. In the interests of the general body of members all societies will exercise this power when it is manifest that the applicant is in very indifferent health, or when there is good ground for the belief that the risk will be one of extra hazard. Now it is impossible to guarantee that a person who is at the present time in robust health will be in the same fortunate position in three months' time. If it were so there would be no need for the National Insurance Act or any other scheme of insurance against sickness. The obvious moral is that delay is dangerous, and entry into insurance as a member of an approved society should be made at the earliest possible moment. The second reason is just as important, though hardly so far-reaching in its consequences.

The procedure necessary to gain admittance as a member of an approved society is very simple, consisting in furnishing satisfactory answers to a few questions, and signing the form in which these answers are set out. This form is technically called the proposal, and it will have to be completed whether entry is made at once or deferred. If the application is accepted, the approved society will at once make all arrangements that are necessary for the issue of the insurance cards and books, thus relieving the member of all trouble in the matter. If entry is deferred the insured person will have to obtain a card from somewhere, before the first payment of wages is made after the Act comes into operation, and the only course open is to apply to a post office for it. The card will only be supplied from the post office after a special form of application has been completed, and this is all extra trouble that may be so easily avoided by applying direct to an approved society for membership.

#### The Consequences of Non-Compliance.

With the approach of July 15 has come a recrudescence of the threats to set the law at defiance, and to resist the payment of contributions, euphemistically termed the "Servant Tax" in certain quarters. Without expressing an opinion on the measure politically we feel bound to call attention to the grave consequences of any such course of action. The Act provides, in a penalty clause, that a fine not exceeding £10 will be incurred, on summary conviction, for every offence against the Act, and if that offence is of the nature of the failure of the employer to pay the contributions to which he (or she) is liable, the employer will, in addition be required to pay the contributions that should have been paid, without the right to recover the employee's portion. This is sufficiently onerous, but if the insured person is a member of an approved society, and has through the neglect or failure of the employer to pay the contributions suffered loss of benefit, the employer will have to make good such benefits as have been lost. It seems fairly clear from the attitude of the Government that it is intended the law should be rigidly obeyed, and it is therefore important that all concerned should bear in mind the consequences of non-compliance.

#### Persons who must be Insured.

In order that our readers may not have occasion for offence against the Act through being unacquainted with its provisions we recapitulate the general principle that all employed persons between the ages of sixteen and seventy must be insured under the Act, if they earn at the rate of not more than £160 a year, and those who work with their hands for an employer must be insured even if they earn more than this. This general rule applies to persons of either sex, whether British subjects or not, but there are some exceptions and modifications. The exceptions are set out at length on the second page of the "N.H. Leaflet," which may be obtained for the asking at any post office. We strongly advise all our readers who may be in doubt as to their position to obtain and carefully read this official leaflet. There are in addition to those who will be exempted from insurance in all cases, two classes of persons, to whom exemption may be granted if they claim to be exempted. These classes are: (1) Those who can show that they have a pension of £26 a year or more, or an income of £26 a year or more which they do not earn by their personal exertions, and (2) those who are ordinarily and mainly dependent for their livelihood upon some other person. Although such persons are exempted from becoming compulsory "employed contributors," their employers will have to pay the employee's contribution, which, but for the special exemption, he would have been required to pay. Forms of application for exemption may be obtained from any post office.

### The Insurance of Charwomen.

There has been much discussion as to whether charwomen have to be insured, or if they are exempt under the heading of casual labour. The official explanatory Leaflet No. 19, which deals with this subject, is therefore very opportune in its appearance last week. In this leaflet we are told that if a charwoman is employed regularly she must be insured whether she is employed for the purposes of her employer's trade or business or not. If she is employed casually, that is to say for odd jobs, she need only be insured if she is employed for the purposes of her employer's trade or business. Thus, for example, a charwoman who is engaged to come to a private house regularly once a week or once a fortnight must be insured, and charwomen who are engaged by business firms to clean offices, shops, or other business premises must be insured even if they work only one odd day, or part of a day. The leaflet goes on to indicate how the payment of contributions may be arranged in rotation when the charwoman is regularly employed by several different employers in each week. If such an agreement is made a special insurance book must be obtained in which the respective employers must sign their names.

### The Cost of the Benefits.

We have continually heard the remark that the contributions required under the Act are much too high for the benefits promised, and we have been referred by large employers to their own experience in works' clubs and the like. It is surprising that this belief is so widespread, but there is no doubt that much of the opposition to the Act rests upon this foundation. Time alone can show the wisdom of the actuarial estimates of the cost, but in the meantime it may be pointed out that the so-called experiences do not admit of valid comparison with the National Scheme. We know of no club or friendly society that has given identical benefits, or that has made such ample provision, small though it is, for extended sickness or disablement. The Government had at its disposal the best actuarial advice obtainable, and when it is borne in mind that all profits that may accrue from the administration of the Act must be used for the benefit of the insured, and the insured *only*, we can see no valid reason for objection to the Act on the ground of cost to the insured.

### Answers to Correspondents.

### INSURANCE OF DISTRICT AND PARISH NURSES.

An Anxious One.—As you are employed as a parish nurse you will have to be insured, and your employer—presumably the Vicar—will be responsible for seeing that the contribution of 6d. a week is paid. He is empowered to deduct 3d. a week from your wages. The full benefits under the Act can only be obtained by joining an approved society, and as the Nurses' Insurance Society has been formed solely for the benefit of trained female nurses, we strongly advise you to join that society. Address the Secretary, 15 Buckingham Street, Strand, London, W.C.

### INSURANCE OF NURSES IN CHARGE OF HOMES.

Nurse A. G., Warrington.—If you are employed by a committee of management or other authority in your position of charge of a girls' home, and your remuneration does not exceed £160 per annum, you will come within the compulsory provisions of the Act. If you conduct the home on your own account, you would be entitled to become a voluntary contributor, provided your income from all sources does not exceed £160 per annum. The benefits and contributions are the same for all approved societies at the outset. It must be left to you to decide between the claims of different societies. Both societies mentioned in your letter are thoroughly reliable. See reply to "An Anxious One" with reference to the Nurses' Insurance Society.

### INCOME OBTAINED WITHOUT PERSONAL EXERTION.

Mrs. S., Weybridge.—As you are employed as a nurse at a salary under £160 per annum, your employer will have to pay his own contribution of 3d. a week on your behalf in any case, and will also have to see that a further 3d. is paid as your own contribution, unless you obtain a special certificate of exemption. Such a certificate may be obtained from the local pension officer on your furnishing satisfactory evidence as to your private means. Inquiry at your Post Office will elicit the name and address of the pension officer. Insurance under the Act does not affect any further insurances for which you may pay independently.

NOTE.—Questions and Correspondence on all doubtful points are invited, and should be addressed to the Editor, The Hospital, 28 Southampton Street, Strand, W.C., and marked "Insurance."

